# The impact of osteopontin on prognosis and clinicopathology of colorectal cancer patients: a systematic meta-analysis

**DOI:** 10.1038/srep12713

**Published:** 2015-08-03

**Authors:** Mingfei Zhao, Feng Liang, Buyi Zhang, Wei Yan, Jianmin Zhang

**Affiliations:** 1 Department of Neurosurgery, Second Affiliated Hospital, School of Medicine, Zhejiang University, Hangzhou, Zhejiang 310009, China; 2 Department of Pathology, Second Affiliated Hospital, School of Medicine, Zhejiang University, Hangzhou, Zhejiang 310009, China

## Abstract

Colorectal cancer (CRC) is one of the most frequent malignant neoplasms worldwide. Up to now, no biomarker has been used to predict the prognosis and surveillance of patients with CRC. Recently, the association between osteopontin (OPN) overexpression and the prognosis of CRC was investigated widely, but the results were inconsistent. Therefore, the aim of present meta-analysis was to assess the prognostic effect of osteopontin in patients with CRC. PubMed, EMBASE, Web of Science, Scopus and Chinese Medical Database were systematically searched. A total of 15 studies containing 1698 patients were included in our meta-analysis. The pooled data of studies showed that high OPN expression was significantly associated with high tumor grades (OR = 2.24, 95% CI 1.55–3.23), lymph node metastasis (OR = 2.36, 95% CI 1.71–3.26) and tumor distant metastasis (OR = 2.38, 95% CI 1.01–5.60). Moreover, high OPN expression was significantly associated with the 2-year (HR 1.97, 95% CI 1.30–3.00), 3-year (HR 1.82, 95% CI 1.24–2.68), 5 year (HR 1.53, 95% CI 1.28–1.82) survival rates and overall survival (OS, HR 1.70, 95% CI 1.12–2.60), respectively. These results indicated that OPN could serve as a prognostic biomarker and as a potential therapeutic target for CRC.

Colorectal cancer (CRC) is one of the most frequent malignant neoplasms worldwide and also one of the leading causes of cancer-related mortality^1^. Though early diagnosis and clinical treatment could improve the prognosis of CRC patients, patients with distant metastasis, remains very poor. So it is essential for us to find a new molecular marker that could predict and improve the prognosis and reduce the mortality of patients with CRC.

Osteopontin (OPN) is a multifunctional phosphoprotein, which could be secreted by a variety of cells, such as lymphocytes, macrophages and osteoclasts^2^,^3^,^4^. Recent studies reported that OPN overexpression has been detected in many human carcinomas, for example, lung cancer, breast cancer, gastric cancer, hepatocellular carcinoma, colorectal cancer^5^,^6^,^7^,^8^,^9^ and so on. It is suggested that OPN levels in blood or tumor samples may be valuable for predicting the prognosis of carcinomas, and the inhibition of OPN might be helpful for the treatment of patients with carcinoma. But the association of OPN overexpression with the prognosis of CRC was not clear, so the objective of present meta-analysis was to determine the possible role of OPN expression in the progression and prognosis of CRC patients.

## Materials and Methods

### Literature search and study selection

PubMed, EMBASE, Web of Science, Scopus and Chinese Medical Database were searched to identify the potential articles related to CRC and OPN up to September 30, 2014. The search of published articles was undertaken using the following terms: “opn”, “osteopontin”, “spp1”, “colorectal”, “colon” and “rectal”.

The results were only from English or Chinese articles. The reference lists and all retrieved articles were also screened for additional relevant articles. The studies collected in present meta-analysis should meet the following criteria: (1) the patients with colorectal cancer were confirmed; (2) the OPN expression levels of the patients were measured; (3) the association of OPN expression with tumor grades, recurrences or survival was evaluated; (4) the sufficient data were provided for estimating the hazard ratios (HRs) or odds ratios (ORs) and their 95% confidence intervals (95% CIs); (5) the articles were written in English or Chinese. For duplicate articles with identical or overlapping data, only the one with biggest sample size or the most recent one was included in the meta-analysis. Conference abstracts, case reports, reviews, editorial letters, abstracts and comments were excluded.

### Data extraction and Quality Assessment

Two investigators (Mingfei Zhao and Feng Liang) independently extracted data from the relevant articles. Disagreement between two investigators was settled through discussion until consensus was reached. Table 1 indicated that the names of authors, years of publication, number of patients, assay methods, sample source, tumor grade, survival rate, OPN expression levels and cutoff value were retrieved from each publication. The Newcastle–Ottawa Scale with several modifications was used to access the quality of the included studies in Table 2^10^. The scale was categorized into three main dimensions: the patient selection, the study comparability and the outcome. Studies with NOS scores >6 were considered to be high quality.

### Statistical analysis

Odds ratios (ORs) with 95% CI were utilized to evaluate the association of OPN expression levels with clinical parameters, while the hazard ratios (HRs) with 95% CI were utilized to analyze the association of OPN expression levels with survival rates. The association of OPN expression levels with overall survival (OS) was also evaluated by HRs with 95% CI. Subgroup analysis was also performed to evaluate the differences between ORs and HRs in collected studies with different detection methods, i.e., immunohistochemistry (IHC), reverse transcription-polymerase chain reaction (RT-PCR) or enzyme-linked immunosorbent assay (ELISA). Heterogeneity of pooled results was assessed using Cochrane’s Q test and *I*^*2*^ measurement. *P* > 0.10 or *I*^*2*^ < 50% indicated that the heterogeneity was not significant, and then a fixed-effects model was used. Otherwise, a random effect model was used. Sensitivity analysis was conducted to evaluate the validity and reliability of present meta-analysis. Begg’s funnel plot and Egger’s test were used to evaluate the publication bias risk. All statistical analyses were performed using STATA version 11.0 (STATA Corporation, College Station, TX, USA), and all *P* values were two sides.

## Results

### Studies selection and the characteristics of the included studies

**Figure 1:**
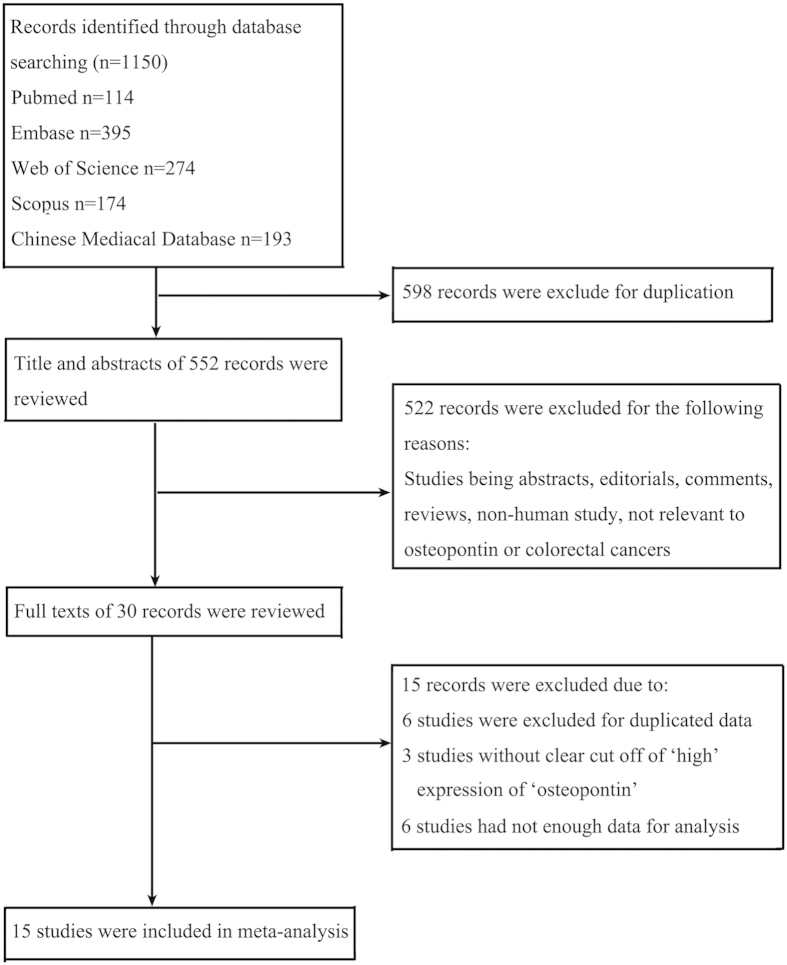
Flow chart of study selection procedure.

A total of 1150 studies were identified using the strategy described above. The detailed screening process was shown in Fig. 1. After careful screening of the titles and abstracts, 30 articles were retained. After reviewing the full text, 15 studies were excluded due to the duplicated data or insufficient data. At last, 15 studies^9^,^11^,^12^,^13^,^14^,^15^,^16^,^17^,^18^,^19^,^20^,^21^,^22^,^23^,^24^ were included in our meta-analysis according to the inclusion criteria (Fig. 1). A total of 1698 patients were included, containing 844 patients (49.7%) with ‘high’ OPN. The basic characteristics of the included studies are shown in Table 1.

### OPN expression and clinicopathological features

**Figure 2:**
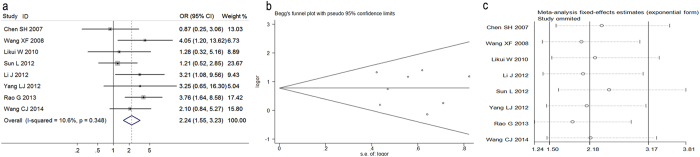
(**a**) Forest plot for the relationships between Osteopontin (OPN) expression and tumor grades of colorectal cancer. (**b**) Begg’s funnel plots of publication bias for meta-analysis of OPN. (**c**) Sensitivity analysis for meta-analysis of OPN.

In Fig. 2a, the pooled data of eight studies showed that high OPN expression was significantly associated with tumor grades (OR = 2.24, 95% CI = 1.55–3.23), and no significant heterogeneity between studies were observed (*I*^*2*^ = 10.6%, *P* = 0.348). Figure 2b indicated there was no significant publication bias (*P* = 0.871). The sensitive analysis was performed by removing studies one by one, and it was found that removal of any individual study did not alter the overall trend, suggesting that the results in present meta-analysis were statistically robust (Fig. 2c).

**Figure 3:**
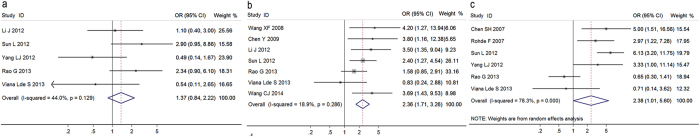
Forest plot for the relationships between Osteopontin (OPN) expression and the tumor stage. (**a**) the relationship between OPN expression and the depth of tumor invasion. (**b**) the relationship between OPN expression and the lymph node metastasis. (**c**) the relationship between OPN expression and the distant metastasis.

Five studies reported the association of the depth of tumor invasion with OPN expression. In Fig. 3a the results of pooled analysis showed no significant association between OPN expression and the depth of tumor invasion (OR = 1.37, 95% CI = 0.84–2.22) and no significant heterogeneity (*I*^*2*^ = 44.0%, *P* = 0.129). Seven studies demonstrated the association of OPN expression with lymph node metastasis. The combined data of the included studies showed the significant association between high OPN expression and lymph node metastasis (OR = 2.36, 95% CI = 1.17–3.26), and no heterogeneity between studies (*I*^*2*^ = 18.9%, *P* = 0.286) in Fig. 3b. Six studies reported the association between OPN expression and tumor distant metastasis in Fig. 3c, there was correlation between high OPN expression and tumor distant metastasis (OR = 2.38, 95% CI = 1.01–5.60), but there was significant heterogeneity among these studies (*I*^*2*^ = 73.8%, *P* = 0.000). The results of Begg’s funnel plot and Egger’s test showed no publication bias in Fig. S1. Sensitivity analysis was also conducted and the overall trend was not changed when the studies were removed one by one (Fig. S2).

### OPN expression and survival rates of patients with CRC

**Figure 4:**
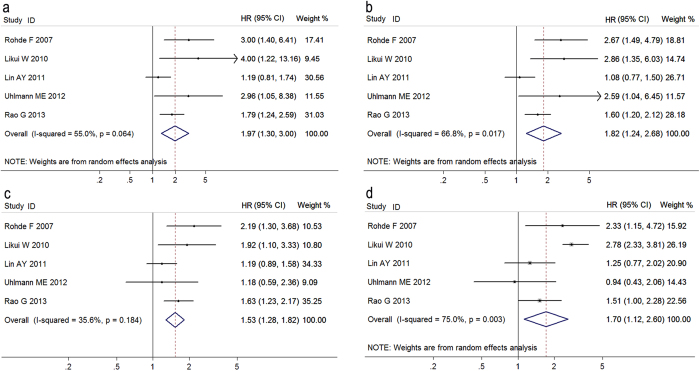
Forest plots for the relationships between osteopontin expression and prognosis of patients with colorectal cancer. (**a**) 2-year survival rate, (**b**) 3-year survival rate, (**c**) 5 year survival rate, d overall survival rate.

Fig. 4 exhibited that high OPN expression was significantly associated with the 2-year (HR 1.97, 95% CI 1.30–3.00), 3-year (HR 1.82, 95% CI 1.24–2.68), and 5 year (HR 1.53, 95% CI 1.28–1.82) survival rates in colorectal cancer patients, respectively. In addition, five studies contained sufficient data for analyzing the relationship between OPN expression and overall survival (OS) in patients with colorectal cancer. There was significant association of high OPN expression with OS (HR 1.70, 95% CI 1.12–2.60), but there was significant heterogeneity (*I*^*2*^ = 75.0%, *P* = 0.011). Begg’s funnel plot and Egger’s test were performed, and no publication bias was found in Fig. S3. Sensitivity analysis was conducted and the overall trend was not changed by removing the studies one by one in Fig. S4.

### Subgroup analysis based on different detection methods

A subgroup analysis on the basis of different detection methods was performed to assess the association of OPN expression with tumor grade and survival rates respectively, owing to the various OPN detection methods used in the included studies. Fig. S5a determined that tumor grade was correlated with OPN expression detected by IHC (OR 2.33, 95% CI 1.60–3.41). Moreover, OPN expression detected by IHC or RT-PCR was significantly related to 5-year survival rate and overall survival of patients with CRC in Fig. S5b and Fig. S5c.

## Discussion

CRC is one of the most prevalent cancers worldwide. Recently the mortality of CRC has decreased significantly owing to the progression in screening of CRC, but till now the prognosis of patients with advance tumor remains very poor^25^. Some clinicopathological parameters, such as carcinoembryonic antigen (CEA) and CA19–9, have been used for screening and monitoring CRC^26^,^27^; however, these parameters remain controversial for predicting the prognosis and surveillance of CRC patients. Thus, it is necessary for us to identify novel molecular biomarkers for predicting the development and prognosis of patients with CRC.

OPN, a secreted multifunctional glyco-phosphoprotein, was identified to play a key role in tumorigenesis, progression and prognosis of a variety of malignant tumors^5^,^6^,^7^,^8^,^9^. Many studies have reported the association between OPN expression and CRC, but results in these studies were not uniform^9^,^11^,^12^,^13^,^14^,^15^,^16^,^17^,^18^,^19^,^20^,^21^,^22^,^23^,^24^. The aim of present meta-analysis was to evaluate the prognosis value of OPN in patients with CRC.

Our meta-analysis includes 15 studies with 1698 CRC patients, several clinicopathological features were significantly associated with OPN expression. Firstly, the results of pooled analysis on the relationship of OPN expression with CRC tumor grade suggested the significant association between high OPN expressions and the high tumor grade. Secondly, the association of OPN expression with the depth of tumor invasion was analyzed. An evident trend toward higher OPN expression with the deeper tumor invasion was identified, though there was no statistically significant difference. Also the results of present meta-analysis showed that high OPN expression was closely related to tumor metastasis, including lymph node metastasis and distant metastasis. Taken together, the pooled data of present meta-analysis supported the hypothesis that high OPN expression might promote CRC invasion and metastasis, leading to the poor prognosis of patients with CRC. Thirdly, the association of high OPN expression with survival of patients with CRC was evaluated. OPN expression was significantly associated with the 2-year, 3-year, and 5-year survival rates, respectively. And the OS was markedly shorter in patients with OPN overexpression. The subgroup analysis stratified by three different detection methods demonstrated that OPN expression detected by IHC was significantly associated with tumor grade, OPN expression detected by IHC or RT-PCR was correlated with both 5-year survival rate and overall survival in patients with CRC. Therefore, OPN can serve as a biomarker for CRC prognosis, and the measurement of OPN expression in CRC patients could be helpful for guiding the clinical treatment of CRC patients.

There are some possible limitations in the present meta-analysis. Firstly, the sample size of studies included in this meta-analysis was relatively small. Secondly, the difference in the cutoff levels of OPN expression among diverse studies may impact on the accurate estimation of prognosis for CRC, because different cutoff of OPN expression may lead to diametrically opposite results. For example, Likui W *et al.*^9^ found OPN expression detected by RT-PCR was an independent prognostic factor for the prognosis of CRC patients (*P* = 0.008). While another study using the same detection method indicated that OPN expression was not a good biomarker for the prognosis of CRC patients (*P* = 0.092)^28^. Although the latter study^28^ only focused on the stage II colon cancer which might be a possible explanation for this difference, the different cutoff of OPN expression may an important interference factor. In future, a large multicenter study using the same detection method and cutoff of OPN expression may be helpful to obtain more accurate results. Thirdly, there are clinical and genetic differences between colon tumors and rectum tumors. However, there are only two studies focused on the colon cancer^11^,^13^, other 13 studies in present meta-analysis did not stratified by tumor sites^9^,^12^,^14^,^15^,^16^,^17^,^18^,^19^,^20^,^21^,^22^,^23^,^24^, and this may be the source of heterogeneity.

In conclusion, the results of present meta-analysis demonstrated that OPN expression might be significantly associated with tumor grade, invasion, metastasis, and survival of CRC patients, although the cut-off value of high OPN should be further studied. Moreover, the Elisa assay of blood may be the best way for OPN detection, which is a relatively noninvasive, objective and accurate assay without operation. OPN can be used to evaluate clinic-pathology of tumor in preoperative and for the surveillance of tumor recurrence postoperative. However, additional large prospective studies with optimal cut-off values of high OPN should be performed to determine our findings in future.

## Additional Information

**How to cite this article**: Zhao, M. *et al.* The impact of osteopontin on prognosis and clinicopathology of colorectal cancer patients: a systematic meta-analysis. *Sci. Rep.* **5**, 12713; doi: 10.1038/srep12713 (2015).

## Supplementary Material

Supplementary Information

## Figures and Tables

**Table 1:** Characteristics of studies for association between OPN and colorectal cancers.

Study/Year (Reference)	Tumor site	Sample size	Sample source	Tumor grade (I–II/III–IV)	OPN Detection method	cut-off level of ‘high’ OPN expression	No. of patients with ‘high’ OPN	Follow-up duration (months)	Survival rate (%)						Study quality (Points)
									2-year		3-year		5-year		
									Low OPN	High OPN	Low OPN	High OPN	Low OPN	High OPN	
Rohde F 2007	colon	120	Tissue	NR	RT-PCR	≥9-fold	39	44–131	88.9	66.7	82.7	53.8	77.8	51.3	8/9
Chen SH 2007	colorectal	60	Tissue	20/40	IHC	>10%	40	NR	NR	NR	NR	NR	NR	NR	6/9
Wang XF 2008	colon	60	Tissue	17/43	IHC	IRS ≥ 4	43	NR	NR	NR	NR	NR	NR	NR	6/9
Chen Y 2009	colorectal	76	Serum	NR	Elisa	>157.9 ng/ml	38	NR	NR	NR	NR	NR	NR	NR	6/9
Likui W 2010	colorectal	84	Tissue	75/9	RT-PCR	>value of 0.276	42	60	92.9	71.4	83.3	52.4	71.4	45.2	7/9
Wild N 2010	colorectal	265	Serum	NR	Elisa	>specificity of 95%	80	NR	NR	NR	NR	NR	NR	NR	6/9
Lin AY 2011	colorectal	154	Tissue	NR	IHC	IRS ≥ 2	90	7–184	60.6	53.3	52.5	48.9	47.5	37.8	8/9
Zhao M 2011	colorectal	30	Tissue	NR	IHC	IRS ≥ 4	23	NR	NR	NR	NR	NR	NR	NR	6/9
Jing LI 2012	colorectal	77	Tissue	57/20	IHC	>10%	38	NR	NR	NR	NR	NR	NR	NR	6/9
Sun L 2012	colorectal	213	Tissue	186/25	IHC	IRS ≥ 2	76	NR	NR	NR	NR	NR	NR	NR	6/9
Yang LJ 2012	colorectal	60	Tissue	17/43	IHC	IRS ≥ 1	43	NR	NR	NR	NR	NR	NR	NR	6/9
Uhlmann ME 2012	colorectal	118	Tissue	NR	RT-PCR	>75% quantile	26	133	92.2	76.9	89.6	73.1	74.0	69.2	8/9
Rao G 2013	colorectal	190	Tissue	138/52	IHC	Moderate staining	124	60	66.7	40.3	54.5	27.4	54.5	25.8	8/9
Viana Lde S 2013	colorectal	114	Tissue	NR	IHC	IRS > 4	103	NR	NR	NR	NR	NR	NR	NR	6/9
Wang CJ 2014	colorectal	76	Tissue	34/42	IHC	IRS > 3	39	NR	NR	NR	NR	NR	NR	NR	6/9

RT-PCR, reverse transcription-polymerase chain reaction; IHC, immunohistochemistry; Elisa, enzyme-linked immunosorbent assay; IRS: immunoreactive score, NR: not reported; OPN: osteopontin.

**Table 2:** Newcastle-Ottawa quality assessment scale.

Selection
(1) Representativeness of the exposed cohort
(a) Truly representative of the average patients with colorectal cancers in the community*
(b) Somewhat representative of the average patients with colorectal cancers in the community*
(c) Selected group of users (e.g., nurses, volunteers)
(d) No description of the derivation of the cohort
(2) Selection of the non exposed cohort
(a) Drawn from the same community as the exposed cohort*
(b) Drawn from a different source
(c) No description of the derivation of the non exposed cohort
(3) Ascertainment of exposure (Proof of colorectal cancers and osteopontin measurement)
(a) Secure record (e.g., surgical records)*
(b) Structured interview*
(c) Written self report
(d) No description
(4) Demonstration that outcome of interest was not present at start of study
(a) Yes*
(b) No
Comparability
(1) Comparability of cohorts on the basis of the design or analysis
(a) Study controls for recurrence or metastasis*
(b) Study controls for any additional factor (Age, gender, grade, KPS score, etc.)*
Outcome
(1) Assessment of outcome
(a) Independent blind assessment*
(b) Record linkage*
(c) Self report
(d) No description
(2) Was follow-up long enough for outcomes to occur? (Death or recurrence)
(a) Yes (60 months)*
(b) No
(3) Adequacy of follow up of cohorts
(a) Complete follow up- all subjects accounted for*
(b) Subjects lost to follow up unlikely to introduce bias-small number lost- (25%) follow up, or description provided of those lost)*
(c) Follow up rate (<75%) and no description of those lost
(d) No statement

A maximum of one star (*)*: can be given for each numbereditem within the ‘Selection’ and ‘Outcome’ categories. While a maximum of twostars**: can be given for ‘Comparability’.
